# Recovery of *Salmonella* isolated from eggs and the commercial layer farms

**DOI:** 10.1186/s13099-017-0223-8

**Published:** 2017-12-14

**Authors:** Mei Long, Hua Yu, Li Chen, Guoyan Wu, Siyue Zhao, Wenwen Deng, Shujuan Chen, Kang Zhou, Shuliang Liu, Li He, Xiaoling Ao, Yubao Yan, Menggen Ma, Hongning Wang, Margaret A. Davis, Lisa Jones, Bei Li, Anyun Zhang, Likou Zou

**Affiliations:** 1 College of Resources, Sichuan Agricultural University, Chengdu, 611130 People’s Republic of China; 2 Sichuan Entry-Exit Inspection and Quarantine Bureau, Chengdu, 610041 People’s Republic of China; 3 National Quality Supervision and Inspection Center of Liquor Products (Sichuan), Yibin, 644000 People’s Republic of China; 4 College of Food Science, Sichuan Agricultural University, Ya’an, 625014 People’s Republic of China; 5 College of Life Sciences, Sichuan University, Chengdu, 610065 People’s Republic of China; 6 College of Veterinary Medicine, Washington State University, Pullman, USA; 7 Dujiangyan Campus, Sichuan Agricultural University, Chengdu, 611830 People’s Republic of China

**Keywords:** *Salmonella*, Environment, Eggs, Production chain, Layer farm

## Abstract

**Background:**

*Salmonella* is recognized as a common bacterial cause of foodborne diarrheal illness worldwide, and animal or its food products have been the most common vehicles of the *Salmonella* infections. This study aimed to investigate the distribution of *Salmonella* in two commercial layer farms and to determine the genetic relatedness between these strains. The *Salmonella* isolates were serotyped by slide agglutination using commercial antisera and analyzed for genetic relatedness using pulsed-field gel electrophoresis (PFGE).

**Results:**

The internal environment had the highest prevalence of *Salmonella* (14/15, 93.3%), followed by external environment (60/96, 62.5%) and egg samples (23/84, 27.3%). The prevalence of *Salmonella* in the environment was significantly higher than that in egg samples (*p* < 0.05). The occurrence of *Salmonella* in the internal environment (93.3%) was relatively higher than in the external environment (55.6–77.2%). The 111 isolates were distributed among 15 PFGE types, and the PFGE results suggested that there existed cross-contamination between these strains not only from eggs, but also from the environments.

**Conclusions:**

The findings indicated ongoing *Salmonella* cross-contamination inside or outside of the layer farms, and that *Salmonella* could also spread along the egg production line.

**Electronic supplementary material:**

The online version of this article (10.1186/s13099-017-0223-8) contains supplementary material, which is available to authorized users.

## Background

*Salmonella enterica* is recognized as one of the most common bacterial cause of human diarrheal illness worldwide, which has been a considerable burden to public health and economic loss. There are 1.4 million occurrences of human salmonellosis in the USA annually, 95% of which were foodborne [1]. Accordingly, *Salmonella*-related infections have been associated with $365 million in annual direct medical costs [2]. In the European Union, 99,020 food-borne cases caused by *Salmonella* were reported in 2010 [3]. The estimated incidence of *Salmonella* gastroenteritis in East Asia circa 2006 was 3980 cases per 100,000 person-years (compared to a global incidence of 1140 per 100,000 person-years) [4]. *Salmonella* outbreaks are commonly associated with consumption of contaminated food, such as poultry meat and eggs, which have been identified as the important vehicle for human salmonellosis [5, 6].

*Salmonella* has been frequently recovered from laying hen house environments, suggesting that the environment of the poultry farm can act as a reservoir for *Salmonella* and contribute to the horizontal dissemination of *Salmonella* via animal-to-animal contact and contaminated feed [7–9]. In addition to feed, the water, feces, dust, cages and litter contaminated with *Salmonella* are important sources of infection [9–15]. Many studies focused on the distribution of *Salmonella* among different sample origins in poultry environments, or on antibiotic resistance, virulence, and control strategies [9, 14, 16, 17]. However, there have been few investigations of the association between *Salmonella* isolates recovered from the internal and external poultry environment and the relationship between isolates obtained from sequential points along the production chain. Recognition of these aspects is important in controlling the spread of *Salmonella* and reducing the prevalence of *Salmonella* in production settings.

Although all serotypes may be regarded as potential human pathogens, the majority of infections are caused by a very limited number of serotypes, of which *Salmonella* Enteritidis and *Salmonella* Typhimurium are the two most common ones associated with gastrointestinal disease of humans [18, 19].

There have been increasing concerns over the past 30 years about the worldwide emergence of multi-drug resistant phenotypes among *Salmonella* serotypes, in particular *S.* Typhimurium. Other non-typhoidal *Salmonella* serovars, such as *S*. Braenderup, *S*. Derby, *S*. Jerusalem and *S*. Bovismorbificans [15, 20], have caused outbreaks but they do not frequently occur or rarely outbreaks. Several serotypes can colonize the digestive tract of chickens and be excreted in feces, which can persist in the environment and may lead to vertical and horizontal transmission in chickens, ultimately contaminating the processing chain and retail poultry products [18].

Previous studies have investigated the distribution and prevalence of *Salmonella* in broiler chickens and the processing environments [9, 14]. In Guangdong Province, China, the most frequent serotypes isolated from raw retail poultry meat were *S.* Enteritidis, *Salmonella* Indiana and *S.* Typhimurium [21] and from live chickens, the most frequent serotypes were unidentified, followed by *S.* Typhimurium and *S.* Enteritidis [22]. However, very little is known about the distribution and relatedness of *Salmonella* from layer farms and the variation of that distribution along the production processing chain, particularly in China. Therefore, the objective of this study was to investigate the distribution of *Salmonella* at each particular link in the internal and external environments of two commercial layer farms and to analyze the relatedness of the prevalent strains along with the egg production chain.

## Methods

### Sampling farms

Sampling was conducted on two layer farms, one built in 1999 and the other in 2006, which both belong to the same commercial egg production company and are 5 km apart. The old layer farm has a capacity for 250 thousand birds, while the new layer farm can hold 500 thousand birds. Additionally, the technical characteristics of the old layer farm are different from those of the new layer farm. Due to the construction time, the facilities of the old layer farm are relatively outdated, and the new layer farm has modern farming technology with fully automated management, egg harvesting, egg washing, disinfection, ultraviolet (UV) irradiation and packaging.

### Egg collection

Eighty-four egg samples were collected from three main parts of the production chain, including cages (placed at the front, middle, and posterior of the internal henhouse) of the old layer farm, egg belt (in the front, middle, and posterior of the belt) and egg collection conveyer of the new layer farm. In addition, retail eggs traced to their farm of origin were collected from the supermarket within their original packaging. All eggs were aseptically transferred into individual sterile plastic bags and transported to the laboratory for cultivation and isolation within 6 h.

### Environmental sample collection

The environmental sampling sites included all major points of the egg processing line and environments, which can be divided into two main parts, the internal and external environments. These sampling sites included disinfectant system, soil, feces, dust and gutter samples of the outdoor environment, and wet curtain cooling system, cage, egg nest samples of the indoor environment of the old layer farm. Samples from the washing room, washing water, irradiation room, package room, storage room and feces of the outdoor environment, and cage, egg belt and egg collection conveyer samples of the indoor environment were collected in the new layer farm (Tables 1 and 2). A total of 126 environmental samples were collected using a sterile cotton swab. After collection, all samples were transported to the laboratory in an ice chest and stored at a 4 °C cooler for bacterial isolation within 6 h.

**Table 1:** The prevalence and distribution of *Salmonella* in the old layer farm

Origins	N^a^	*Salmonella* serotypes						Prevalence^b^
		*S*. Derby	*S*. Braenderup	*S*. Enteritidis	*S*. Jerusalem	*S.* Bovismorbificans	Un-identified	
External environment of the henhouse								
Disinfection system	5	1						20.0% (1/5)
Soil	15	5		3	2			66.7% (10/15)
Feces	13	11			1			92.3% (12/13)
Dust	6	5						83.3% (5/6)
Gutter	3	1					1	66.7% (2/3)
Internal environment of the henhouse								
Wet curtain cooling system	3	2				1		100% (3/3)
Cage	6	5						83.3% (5/6)
Egg nest	6	6						100% (6/6)
Total	57	36		3	3	1	1	77.2% (44/57)

^a^Sample number

^b^The prevalence of *Salmonella* was among the samples in different sampling site

**Table 2:** The prevalence and distribution of *Salmonella* in the new layer farm

Origins	N^a^	*Salmonella* serotypes						Prevalence^b^
		*S*. Derby	*S*. Braenderup	*S*. Enteritidis	*S*. Jerusalem	*S.* Bovismorbificans	Un-identified	
External environment of the henhouse								
Washing room	12	1			1			16.7% (2/12)
Washing water	11	2			3	5	1	100% (11/11)
Irradiation room (UV)	1				1			100.0% (1/1)
Package room	9	3						33.3% (3/9)
Storage room	12				3		1	33.3% (4/12)
Feces	9	4		1	2	1	1	100.0% (9/9)
Internal environment of the henhouse								
Cage	6	6						100% (6/6)
Egg belt	6	6						100% (6/6)
Egg collection conveyer	3	1			1			66.7% (2/3)
Total	69	23		1	11	6	3	63.8% (44/69)

^a^Sample number

^b^The prevalence of *Salmonella* was among the samples in different sampling site

### *Salmonella* enrichment

Modified enrichment methods were used based on the preliminary data on *Salmonella* growth from eggs described previously [18]. Briefly, a swab technique was used to sample intact egg surfaces. Swabs were directly inoculated into 10 mL Buffer Peptone Water (BPW) for pre-enrichment and incubated at 37 °C for 18–24 h. After eggshell surface processing, each eggshell surface was dipped into 3:1 solution consisting of three parts of 70% alcohol to one part iodine tincture solution for 1 min to kill any bacteria on the outside of the shell and was allowed to air dry in a biosafety cabinet. The eggs were cracked open and egg contents were placed into a sterile container. The internal eggshell was washed with sterile phosphate buffered saline (PBS) to rinse off the adhering egg albumen. The internal shell and membranes from eggs were crushed into pieces, transferred to a 250 mL sterile triangular flask with BPW and incubated at 37 °C for 18–24 h. Subsequently, mixed egg content was inoculated into BPW and incubated at 37 °C for 18–24 h. The ratio of egg shell to BPW and content sample to BPW was maintained at 1:10 by volume. The environmental samples were also pre-enriched in BPW at 35 °C for 18–24 h.

### *Salmonella* confirmation

One millilitre of overnight culture was inoculated to 100 mL Rappaport–Vassiliadis (RV) Broth and 10–100 mL Tetrathionate Broth Base (TTB) (Beijing Land Bridge Technology Co, Ltd., Beijing, China) [23] and the inoculated broth was then incubated at 42 °C for 24 h. A full loop of each of the enrichment RV and TTB broth was streaked on Brilliant Green Sulfadiazine agar (BGS) and Xylose Lysine Desoxycholate (XLD) plates, and then incubated at 37 °C for another 24 h. Presumptive *Salmonella* colonies from each plate were stabbed into Triple Sugar Iron agar (TSI) and urea-agar slants (Beijing Land Bridge Technology Co, Ltd., Beijing, China) [24]. After 24 h of incubation at 37 °C. Isolates with typical *Salmonella* phenotypes were confirmed by PCR. The PCR assays for identification of *Salmonella* were previously described [25]. A 284-bp PCR product targeting *inv*A was amplified using primers *inv*A139 (5′-GTGAAATTATCGCCACGTTCGGGCAA-3′) and *inv*A141 (5′-TCATCGCACCGTCAAAG GAACC-3′). Confirmed isolates were stored in TSB containing 20% glycerol at − 80 °C until use. The isolates were further serotyped by slide agglutination using commercial antisera purchased from SSI (Statens Serum Institute, Denmark) according to the manufacturer’s instructions.

### Pulsed-field gel electrophoresis

All isolates were compared using pulsed-field gel electrophoresis (PFGE) analysis according to the PulseNet protocol. The *Xba*I-digested DNA fragments were separated in a 1% agarose gel using a CHEF MAPPER electrophoresis system (Bio-Rad, Hercules, California, USA). The electrophoresis conditions were as previously described [26]. *S. enterica* serovar Braenderup H9812 was used as a marker. PFGE results were analyzed by BioNumerics software (Applied Maths, Kortrijk, Belgium), and banding patterns were compared by using Dice coefficients with a 1.5% band position tolerance.

### Statistical analysis

Frequency differences among the isolates were analyzed using SPSS v.12 (SPSS Inc. 1989–2003), and the Chi square test was used to determine the significance of the differences. A p value less than 0.05 was considered statistically significant.

## Results

### Recovery of *Salmonella* isolates from eggs

A total of 23 *Salmonella* isolates were recovered from 84 egg samples with the prevalence of 27.3%. Among these 23 *Salmonella*, 26.1% (n = 6) were isolated from eggshell surface, 43.5% (n = 10) from internal eggshell and 30.4% (n = 7) from egg content. As shown in Additional file 1: Table S1, retail egg samples had a relatively higher prevalence (n = 6, 50.0%) than those from the internal environment of the new layer farm (n = 10, 33.3%), followed by eggs from the internal environment of the old farm (n = 7, 16.7%). Totally, four serotypes were detected in 23 *Salmonella* isolates from egg samples. *S*. Jerusalem and *S*. Braenderup were the predominant serotype (n = 6, both 26.1%), followed by *S*. Derby (n = 5, 21.7%) and *S*. Bovismorbificans (n = 1, 4.3%), respectively. Additionally, 21.7% (n = 5) isolates was still un-serotyped.

### Frequency of *Salmonella* isolates in layer farm environment

Totally, 88 (69.8%) *Salmonella* isolates were isolated from 126 environmental samples (Tables 1 and 2). Only a slight difference in the prevalence of *Salmonella* was found between the old (44/57, 77.2%) and new (44/69, 63.8%) commercial layer farms (*p* = 0.10, *p* > 0.05). Among all isolates, 68.2% (60/88) were recovered from external samples, while 31.8% (28/88) were detected in internal samples. The internal environment had the highest prevalence of *Salmonella* (28/30, 93.3%), followed by external environment samples (60/96, 62.5%) and the egg samples (23/84, 27.3%). The prevalence of *Salmonella* in environmental samples was higher than in egg samples.

The prevalence of *Salmonella* recovered from the old layer farm is displayed in Table 1. 44 (n = 57, 77.2%) *Salmonella* isolates were recovered from the old layer farm. The incidence of *Salmonella* in external environment of the old layer farm was 71.4% (30/42), and 93.3% (14/15) in internal environment. In external environmental samples, the most frequently observed *Salmonella* contamination was in fecal samples (12/13, 92.3%), followed by dust (5/6, 83.3%), soil (10/15, 66.7%) and gutter samples (2/3, 66.7%). The disinfection system showed the lowest frequency of *Salmonella* (1/5, 20.0%). In the internal environments, the wet curtain cooling system (3/3) and egg nest samples (6/6) had the highest incidence of *Salmonella* (both 100%), followed by cage samples (5/6, 83.3%). Additionally, there were no significant differences between external (71.4%, n = 30) and internal (93.3%, n = 14) environment samples (*p* = 0.34, *p* > 0.05).

Forty-four (n = 69, 63.8%) *Salmonella* isolates were recovered from the new layer farm (Table 2). The incidence of *Salmonella* in external environment of the new layer farm was 55.6% (30/54) and 93.3% (14/15) in internal environment. In external environment samples, washing water, irradiation room (UV) and feces had the highest incidence of *Salmonella* (all 100%), followed by the package and storage rooms (both 33.3%). The washing room had the lowest contamination of *Salmonella* (2/12, 16.7%). However, no significant difference was found between the external (55.6%, n = 30) and internal (93.3%, n = 14) environmental samples (*p* = 0.16, *p* > 0.05).

### Distribution of different *Salmonella* serotypes in layer farm environment

Serotypes detected among the 88 *Salmonella* isolated from environment of the two layer farms included *S*. Derby, *S*. Jerusalem, *S*. Bovismorbificans, *S*. Enteritidis, and unidentified serotypes. The most frequently observed *Salmonella* serovar was *S*. Derby (67.0%, n = 59), followed by *S*. Jerusalem (15.9%, n = 14), *S*. Bovismorbificans (8.0%, n = 7) and *S*. Enteritidis (4.5%, n = 4). Four isolates could not be serotyped (4.5%). Interestingly, *S*. Braenderup was only detected in egg samples, and there were no *S*. Enteritidis in egg samples.

As shown in Table 1, four *Salmonella* serotypes were present among 44 *Salmonella* isolates in the old layer farm. *S*. Derby was most frequently recovered serotype (81.8%, n = 36), followed by *S*. Enteritidis and *S.* Jerusalem (both 6.8%, n = 3), and *S*. Bovismorbificans (2.3%, n = 1). *S*. Jerusalem was found in soil and fecal samples. Interestingly, *S*. Enteritidis was only present in the soil and *S*. Bovismorbificans was detected only in wet curtain system samples. One isolate with an unidentified serotype was recovered from a gutter sample.

Four *Salmonella* serotypes were also found in the new layer farm among the 44 *Salmonella* isolates, of which S. Derby was also the predominant serotype (52.3%, n = 23), followed by *S*. Jerusalem (25.0%, n = 11), *S*. Bovismorbificans (13.6%, n = 6), and *S*. Enteritidis (2.3%, n = 1), respectively. *S*. Derby was found in most samples but absent from irradiation room (UV) and storage room samples. *S*. Jerusalem was present in samples from the egg collection conveyer, washing room, washing water, irradiation room, storage room and feces. *S*. Bovismorbificans was detected in washing water and feces. Strangely, *S*. Enteritidis was only present in one fecal sample. Additionally, unidentified isolates were isolated from washing water, storage room and fecal samples.

### Frequency of *Salmonella* in egg production chain

On the new layer farm, four points of the production chain were sampled (Table 3). Twenty-three of the 45 farm-level samples (51.1%), 27.3% (n = 6) at the processing level, 33.3% (n = 4) at the storage level and 50.0% (n = 6) at the retail level were positive for *Salmonella* contamination. Farm-level samples included those from cage, egg belt, egg (from belt), egg collection conveyer and egg (from conveyer), processing level included washing and irradiation room and package room samples, storage level included the storage room and retail level included retail eggs.

**Table 3:** Different level of the production chain of the two layer farms

Level	Origins	N^a^	*Salmonella* serotypes						Prevalence^b^
			*S*. Derby	*S*. Braenderup	*S*. Enteritidis	*S*. Jerusalem	*S.* Bovismorbificans	Un-identified	
Old layer farm									
Farm	Cage	6	5						33.3% (18/54)
	Egg (from cage)	42	3			3		1	
	Egg nest	6	6						
New layer farm									
Farm	Cage	6	6						51.1% (23/45)
	Egg belt	6	6						
	Egg (from belt)	18	1	3		1	1	1	
	Egg collection conveyer	3	1			1			
	Egg (from conveyer)	12				1	1	1	
Processing	Washing room	12	1			1			27.3% (6/22)
	Irradiation room (UV)	1				1			
	Package room	9	3						
Storage	Storage room	12				3		1	33.3% (4/12)
Retail	Retail eggs	12	1	1		1		3	50.0% (6/12)

^a^Sample number

^b^The prevalence of *Salmonella* was among the samples in different level of the production chain

### PFGE typing

To determine genetic similarity among isolates from different origins, we defined PFGE types as having similarity index equal to or greater than 75%. Overall, a total of 15 distinct PFGE types were identified among the 111 *Salmonella* isolates (Additional file 2: Table S2, Additional file 3: Figure S1). Interestingly, isolates of PFGE type 1 were found in diverse samples including feces, dust, cage, egg nest, egg belt and washing room. Isolates 1 (dust), 2 (feces) from the external environment of the old layer farm had high similarity to isolate 3 (cage) and 4 (egg nest) of the internal environment of the old layer farm as well as to isolates 10, 11, and 12 (cages) from the internal environment and isolates 13 and 14 (both from feces) from the external environment of the new layer farm. The same results were also found in isolate 34, 35 and 36, 37 in different environment of the old layer farm. Additionally, the isolate 23 and 24, 44 and 45 of the old layer farm and 57 and 58, 68, 69 and 70 of the new layer farm in external environment also showed highly similarity to each other. There were also existed genetically relationship between isolates 6, 7 and 8 from internal environment.

The isolates 6, 7 (from egg belt) of the new layer farm and 8 (from egg nest) of the old layer farm both in internal environment samples had been found a highly similarity to each other, respectively. Besides, the isolate 27, 28 (both from feces samples) of the new layer farm had also been found genetically related to the isolate 29 isolated from disinfection room samples of the old layer farm. The same results were also found between the isolates 57 (washing water), 58 (package room) of new layer farm and 59 (soil) of old layer farm as well as 75 (storage room) and 76 (feces), respectively. Indistinguishable *S*. Jerusalem isolates were also from different farms as were the *S*. Jerusalem isolates within type 5. The same results were also found in *S*. Bovismorbificans in type 9.

Additionally, the isolates from egg origins were also related to the *Salmonella* isolates from environment samples of the two layer farms. Two *S*. Braenderup isolates, one (94) isolated from the retail, another (95) from egg samples of belt, had a highly similarity of more than 85%. The same results had also been found in four un-identified isolates (107 and 108, 110 and 111) with different origins.

## Discussion

Human salmonellosis has been consistently associated with the consumption of poultry products worldwide [5, 27]. *S*. Derby was most frequently observed in layer farm environments while *S*. Jerusalem, *S*. Braenderup and *S*. Derby were the predominant serotypes in egg samples. Derby was one of the main serotypes in the present study, but only small outbreaks have been associated with this serotype according to the reports of the CDC website of USA. This finding may be attributable to the inherent physiological characteristics of Derby which lacks pathogenicity islands 13 and 14, the fimbrial *lpf* operon, and other regions that encode metabolic functions [28]. *S.* Jerusalem isolates from a chicken farm have also been reported previously [29]. Braenderup is reportedly a major cause of outbreaks in America [30]. Compared to environmental samples, the incidence of *Salmonella* was lower in egg samples, which was slightly lower (16.7% in old layer farm) or consistent (33.3%) with another report of *Salmonella* isolation from eggshells (34%) [31]. In this study, *Salmonella* isolates were recovered not only from eggshells, but also from egg content. Previous studies revealed that under normal conditions of storage and moisture, *Salmonella* contaminating eggshells could migrate to the egg content [27], which might result in human infections.

Notably, the prevalence of *Salmonella* contamination in environment of the layer farm was somewhat higher than that in eggs in this and other studies [14, 27]. This prevalence was also higher compared with reported prevalence in live broiler chicken samples [14]. The environment of the layer farm was considered as a reservoir for *Salmonella* and could contribute to the horizontal/vertical dissemination of *Salmonella* [14, 32], since *Salmonella* had the ability to persist in both host and non-host environments for its enhanced survival capabilities [33].

Additionally, the incidence of *Salmonella* in the internal environment (93.3%) was somewhat higher than in the external environment (55.6–77.2%) of both layer farms. High similarities between these isolates were also found, which suggested that cage, egg belt and egg nest were the important reservoirs for *Salmonella* in the internal environments and that transmission of *Salmonella* occurs readily between locations in the internal environments. Furthermore, direct contact between egg belt and egg nest eggs were considered to be efficient mechanisms for the transmission of *Salmonella* [9, 11]. Although not assessed in this study, it is plausible that insects and mice play a role as vectors of *Salmonella* in internal environments of layer farms [34, 35]. Contaminated laying hens could spread *Salmonella* to nearby hens by direct contact or could disseminate *Salmonella* in egg forming by its reproductive tract [27]. Thus, these factors indicate complex network of potential cross-contamination of *Salmonella* in the internal environments of layer farms.

There also was high similarity among *Salmonella* isolates from the external environment. Feces, dust, water, and soil were the main source reservoirs for *Salmonella* in the external environments. Feces played an important role in *Salmonella* dissemination, as contaminated feces excreted into the environment could then be a source of the bacteria to naive hosts, perpetuating its survival over the layer farm environment [9, 11, 14, 32]. *Salmonella* was also detected in rinsing water for egg washing. Contaminated washing water flowed along with the gutter and may be used for irrigation water, which could be a major route of *Salmonella* contamination for crops and produce [36, 37]. Dust has also been considered as a vector for *Salmonella* spread through potential airborne transmission [34]. *Salmonella* in dust could also contaminate pelleted feed [34, 38]. Contaminated soil could act as a persistent source of *Salmonella* difficult to disinfect [34, 37]. A moist floor associated with daily rinsing with water for cleaning and the spillage of water from the drinkers could provide favorable condition for survival of *Salmonella* [14].

The difference in prevalence of *Salmonella* in the external environment of the new layer farm to that of the old layer farm (55.6% [30/54] versus 71.4% [30/42]) supports the use of newer farming technology as helpful for controlling *Salmonella* contamination. The lower frequency of *Salmonella* contamination in the disinfection system showed that disinfectant application and washing of eggs was contributing to preventing or reducing bacteria. To prevent *Salmonella* contamination in external environment, litter, feces and dust should be removed frequently and disinfectant applied to surfaces.

*Salmonella* isolates from internal and external environmental samples were also highly similar to each other. This might be explained by cross contamination between internal and external environments when birds or feces are removed [39]. Dust and waste drinking water also have the potential ability to spread *Salmonella* by airborne and waterborne transmission. Other vectors, such as mice, insects and wild birds could introduce *Salmonella* from external to internal environment [40, 41].

The prevalence of *Salmonella* changed dynamically along the egg production chain. The farm level had the highest prevalence of *Salmonella*, followed by retail level, storage and processing level. The reduced recovery of *Salmonella* at processing level might be owing to the strategies for prevention of egg contamination. Egg washing and disinfection were efficient ways to wash bacteria off of egg surfaces. Sealed packages also prevented contamination of eggs. However, during prolonged storage period, the risk of *Salmonella* contamination may increase and result in decreased quality of the egg products. Isolates from individual eggs were genetically similar to each other, which suggested that the *Salmonella* contamination of eggs at the farm level could persist into the retail level. *Salmonella* has been confirmed as having the capacity to colonize the reproductive tract of the laying hens and thereby contaminating forming eggs [34, 42, 43]. We found that isolates from different parts of the production chain were highly similar. Isolates from three different parts of the production chain (farm level, egg production processing part and retail level) had more than 85% similarity. The same results had also been found in isolates 94 (retail level) and 95 (farm level), 110 (retail level) and 111 (storage level). These results indicated that the pathogens could spread along with the poultry breeding to and production chain.

Cross-contamination between two layer farms was also evident from the PFGE results. The most probable explanation could be that breeder chickens upstream of the production chain contaminated both farms. The two layer farms had a same origin of layer chicks, which could introduce *Salmonella* [44]. Contaminated layer hens could transmit *Salmonella* to the forming egg within the reproductive tract [27], which may be the reason the two farms had the same frequency of *Salmonella* contamination (93.3%) in internal environment. Another essential factor might owe to the exchange workers, equipment or managers between two layer farms. Humans and equipment as mechanical vectors could introduce *Salmonella* to each other indirectly [34, 39].

The findings presented herein indicated that there was a significant difference in contamination of *Salmonella* serotypes among egg samples and environmental samples. *S*. Enteritidis was absent in egg samples but present in environmental samples, which was different from previous studies [14, 18]. In general, *S*. Enteritidis was confirmed as strongly associated with shell eggs and egg containing products [45]. The results might be due to strategies applied at the feeding and production processing line, such as disinfection, washing and UV radiation. Disinfection and UV radiation have the capacity to reduce or kill microorganisms on egg shell surfaces [34, 46, 47]. Egg washing was also used to reduce the bacterial contamination and to prevent penetration of bacteria to the egg contents [34]. Additionally, *S*. Braenderup was only present in egg samples, but not in environmental samples. Serotype-specific characteristics may explain their own niche preferences within poultry environments [48, 49].

This study showed that *Salmonella* contamination is common in the layer farms that we studied. *S*. Derby was most frequently observed in layer farm environments while *S*. Jerusalem, *S*. Braenderup and *S*. Derby were the predominant serotypes in egg samples. The prevalence of *Salmonella* in environment of the layer farm was higher than that in egg samples. The incidence of *Salmonella* in internal environment was relatively higher than in external environment in both layer farms. *Salmonella* could be disseminated not only between internal and external environment, but also between different layer farms. It could also spread along the egg production processing chain. Measures, such as cleaning and disinfection routinely etc., should be taken to prevent or reduce the dissemination of *Salmonella* in layer farm environment.

## Conclusions

The findings indicated ongoing *Salmonella* cross-contamination inside or outside of the layer farms, and that *Salmonella* could also spread along the egg production line.

## Additional files

**Additional file 1: Table S1.** The prevalence and distribution of *Salmonella* in egg samples.

**Additional file 2: Table S2.** PEGE type of *Salmonella* in different origins of the two layer farms.

**Additional file 3: Figure S1.** PFGE of *Salmonella* isolates from the two layer farms.

## Abbreviation

PFGE: pulsed-field gel electrophoresis
