# Supplementary material for: Recovery of Salmonella isolated from eggs and the commercial layer farms
Source: Gut Pathog. 2017 Dec 14;9:74. doi: 10.1186/s13099-017-0223-8 (PMC5729242; doi:10.1186/s13099-017-0223-8)
Supplement: Supplementary file 1 — Additional file 1: Table S1. The prevalence and distribution of Salmonella in egg samples. [file 13099_2017_223_MOESM1_ESM.docx]

| **Table S1 The prevalence and distribution of *Salmonella* in egg samples** | | | | | | | | |
| --- | --- | --- | --- | --- | --- | --- | --- | --- |
| **Origins** | **N^A^** | ***Salmonella* Serotypes** | | | | | | **Prevalence ^B^** |
|  |  | *S*. Derby | *S*. Braenderup | *S*. Enteritidis | *S*. Jerusalem | *S.* Bovismorbificans | Un-identified |  |
|  |  | **Old layer farm** | | | | | |  |
| Internal environment | 42 | 3 |  |  | 3 |  | 1 | 16.7%(7/42) |
|  |  | **New layer farm** | | | | | |  |
| Internal environment | 30 | 1 | 5 |  | 2 | 1 | 1 | 33.3%(10/30) |
|  |  | **Market** | | | | | |  |
| Retail eggs | 12 | 1 | 1 |  | 1 |  | 3 | 50.0% (6/12) |
| Total | 84 | 5 | 6 |  | 6 | 1 | 5 | 27.3%(23/84) |

**A**: Sample Number

**B**: The prevalence of *Salmonella* was among the egg samples in different sampling sites.
