# Supplementary material for: Recovery of Salmonella isolated from eggs and the commercial layer farms
Source: Gut Pathog. 2017 Dec 14;9:74. doi: 10.1186/s13099-017-0223-8 (PMC5729242; doi:10.1186/s13099-017-0223-8)
Supplement: Supplementary file 2 — Additional file 2: Table S2. PEGE type of Salmonella in different origins of the two layer farms. [file 13099_2017_223_MOESM2_ESM.docx]

| **Table S2. PEGE type of *Salmonella* in different origins of the two layer farms** | | | |
| --- | --- | --- | --- |
| **PEGE Type**  **(Number of Strains)** | **Farm, Source (n) of isolates** | **PEGE Type**  **(Number of Strains)** | **Farm, Source (n) of isolates** |
| 1 (62) | **O:** Dust (5); Feces(11); Cage(5); Egg nest(6);Disinfection room(1);Wet curtain cooling system(2);Soil(4);Gutter(1); Egg(from cage)(2); **N:** Egg belt(6);Washingroom(1); Cage(5);Feces(4);Egg collection conveyer(1);Package room(3);Egg(from belt)(1);Washing water(2);Cage(1) ; **M:** Retail egg (1) | 4 (13) | **O:** Soil(1);Egg(from cage)(1);Feces(1); **N:** Feces(1);Egg(from belt)(1);Irradiation room(1);Washing water(1);Egg collection conveyer(1);Egg(from conveyer)(1);Storage room(2);Washing room(1); **M:** Retail(1); |
| 2 (1) | **O:** Soil(1) | 3 (1) | **O:** Egg(from cage)(1) |
| 5 (2) | **O:** Soil(1); **N:** Washing water(1) | 6 (1) | **O:** Egg(from cage)(2) |
| 7 (2) | **N:** Washing water(1); Feces(1) | 8 (1) | **N:** Storage room(1) |
| 9 (8) | **O:** Wet curtain cooling system(1) **N:** Washing water(5);Feces(1);Egg(from belt)(1); | 10 (1) | **N:** Egg(from conveyer(1); |
| 11 (4) | **N:** Egg(from belt)(3); **M:** Retail(1); | 12 (2) | **O:** Soil(1); **N:** Feces(1); |
| 13 (2) | **O:** Soil(2) |  |  |
| 14 (4) | **O:** Egg(from belt)(1); **N:** Washing water(1); **M:** Retail(2) | 15 (5) | **O:** Gutter(1); Egg(from cage)(1); **N:** Egg(from conveyer)(1);Feces(1);Storage room (1); **M:** Retail(1); |

Note: “O” , Old layer farm, “N”, New layer farm, and “M”, Market
