# Supplementary material for: Recovery of Salmonella isolated from eggs and the commercial layer farms
Source: Gut Pathog. 2017 Dec 14;9:74. doi: 10.1186/s13099-017-0223-8 (PMC5729242; doi:10.1186/s13099-017-0223-8)
Supplement: Supplementary file 3 — Additional file 3: Figure S1. PFGE of Salmonella isolates from the two layer farms. [file 13099_2017_223_MOESM3_ESM.docx]

Ex

Ex

Ex

In

In

Ex

Ex

In

Ex

In

Ex

In

Ex

In

Ex

Ex

Ex

Ex

In

In

Ex

In

In

Ex

In

In

In

Ex

Ex

In

In

In

In

In

In

In

Ex

Ex

Ex

In

In

Ex

Retail

Origin

No.

Serotype

Farm

Type

Environment

Ex

Ex

Ex

Ex

Ex

In

Ex

Ex

In

Ex

In

In

Ex

Ex

In

Ex

Ex

Ex

Ex

Ex

In

Ex

Ex

In

Ex

In

Ex

Ex

Ex

In

Ex

In

Retail

Ex

Ex

Ex

Ex

Ex

In

In

Ex

Ex

Ex

Ex

Ex

Ex

Ex

Ex

In

Ex


**Fig. S 1 PFGE of *Salmonella* isolates from the two layer farms**

In

In

Ex

In

In

Retail

In

Ex

Ex

Ex

Ex

In

Ex

Retail

Retail

Ex

In

In

Ex

Retail

Ex

In
